# CD4^+^ T cell immunity to *Salmonella* is transient in the circulation

**DOI:** 10.1371/journal.ppat.1010004

**Published:** 2021-10-25

**Authors:** Newton G. Peres, Nancy Wang, Paul Whitney, Sven Engel, Meghanashree M. Shreenivas, Ian Comerford, Dianna M. Hocking, Anna B. Erazo, Irmgard Förster, Andreas Kupz, Thomas Gebhardt, Shaun R. McColl, Stephen J. McSorley, Sammy Bedoui, Richard A. Strugnell

**Affiliations:** 1 Department of Microbiology & Immunology, The University of Melbourne at the Peter Doherty Institute for Infection and Immunity, Melbourne, Australia; 2 European Molecular Biology Laboratory Australia Node for Single Molecule Science, School of Medical Sciences, The University of New South Wales, Kensington, Australia; 3 School of Biological Science, Faculty of Sciences, The University of Adelaide, South Australia, Australia; 4 Immunology & Environment, Life and Medical Sciences (LIMES) Institute, University of Bonn, Bonn, Germany; 5 Centre for Molecular Therapeutics, Australian Institute of Tropical Health and Medicine, James Cook University, Cairns, Australia; 6 Center for Comparative Medicine, University of California, Davis, California, United States of America; Stanford University School of Medicine, UNITED STATES

## Abstract

While *Salmonella enterica* is seen as an archetypal facultative intracellular bacterial pathogen where protection is mediated by CD4^+^ T cells, identifying circulating protective cells has proved very difficult, inhibiting steps to identify key antigen specificities. Exploiting a mouse model of vaccination, we show that the spleens of C57BL/6 mice vaccinated with live-attenuated *Salmonella* serovar Typhimurium (*S*. Typhimurium) strains carried a pool of IFN-γ^+^ CD4^+^ T cells that could adoptively transfer protection, but only transiently. Circulating *Salmonella*-reactive CD4^+^ T cells expressed the liver-homing chemokine receptor CXCR6, accumulated over time in the liver and assumed phenotypic characteristics associated with tissue-associated T cells. Liver memory CD4^+^ T cells showed TCR selection bias and their accumulation in the liver could be inhibited by blocking CXCL16. These data showed that the circulation of CD4^+^ T cells mediating immunity to *Salmonella* is limited to a brief window after which *Salmonella*-specific CD4^+^ T cells migrate to peripheral tissues. Our observations highlight the importance of triggering tissue-specific immunity against systemic infections.

## Author summary

Helper T cells are essential for controlling infections by bacterial pathogens, such as *Salmonella enterica* var Typhimurium (*S*. Typhimurium). While it is well-established that this role is related to their provision of IFN-γ, when and where helper T cells elicit their protective function in vivo remains unresolved. We identified a protective helper T cell population in the circulation of mice early after inoculation with growth-attenuated *S*. Typhimurium strains; this population waned overtime. We observed that circulating helper T cell immunity can adoptively protect naïve recipient mice against lethal *S*. Typhimurium infection when harvested from a short time-window. In comparing helper T cell responses between spleen and liver in *Salmonella*-infected mice, we have observed a previously uncharacterized trafficking of helper T cells to the liver followed by the residence of *S*. Typhimurium-specific T cell memory in the organ. Taken together these findings identify that protective immunity to *Salmonella* infections is transient in the circulation and the liver as a preferential site of helper T memory cells.

## Introduction

The facultative intracellular pathogen *Salmonella enterica* causes infections that range from self-limiting gastroenteritis to life-threatening conditions, such as typhoid fever. Typhoid fever is characterized by rapid bacterial dissemination, resulting in septicemia and central nervous system infections with potentially fatal outcomes [1,2]. Treatments for *Salmonella* infection remain limited and the rise of antibiotic resistance is likely to further reduce viable therapy options. Thus, enhancing the immunological status via immunization may assist in the control of *Salmonella* infections in endemic regions. Murine infections with *Salmonella enterica* serovar Typhimurium (*S*. Typhimurium) have served as a key instrument for studying the immune response to *Salmonella*. The wild type strain SL1344 is highly virulent in susceptible mouse strains, where it rapidly disseminates and causes systemic disease to which C57BL/6 mice, for example, succumb within 5–7 days [3]. Growth-attenuated *S*. Typhimurium strains, in contrast, can be controlled by the immune system, providing the host with protective immunity against re-infection with SL1344 [4,5]. Multiple arms of the immune system contribute to immunity against *Salmonella* infections [6–8]. Innate sensors and their respective signaling pathways provide inflammatory cues that facilitate the priming of adaptive responses, amongst which CD4^+^ T cells and IFN-γ play vital roles [4,9]. Resistance to re-infection also depends heavily on CD4^+^ T cells, IFN-γ and/or IL-12 [4,10], highlighting the overall importance of T helper 1 (Th1) immunity in controlling this intracellular pathogen during primary and secondary infections.

Attempts at characterizing the protective *Salmonella*-specific CD4^+^ T cell response have yielded some seemingly puzzling observations. The CD4^+^ T cells that respond early to *Salmonella* infection include T cells specific for epitopes derived from the FliC subunit of flagellin [11]. Although flagellated bacteria are present in the spleen, and dendritic cells isolated from the infected spleen can present FliC-derived antigen in the context of MHC class II molecules, activated flagellin-specific CD4^+^ T cells are remarkably low in frequency, if not in the spleen [11]. Moreover, adoptive transfer of memory CD4^+^ T cells isolated from spleen or lymph nodes of mice previously infected with a growth-attenuated strain failed to confer protection to recipients [10]. In human volunteer studies, immunization with live-attenuated vaccines against serovar Typhi identified *Salmonella*-reactive Th1 cells in the bloodstream 1–3 weeks after vaccination [12], followed by minor reactivity in the circulating memory pool [13]. Thus, although *Salmonella*-specific CD4^+^ T cells clearly play a vital role in immunity against *Salmonella* [4,9,10], those that confer protection likely distribute to tissues beyond the circulation and secondary lymphoid organs, such as spleen and lymph nodes. CD4^+^ T responses to systemic *Salmonella* Typhimurium demonstrate a level of organ specificity and assume distinctive trafficking behaviors to fulfill complex adaptive responses [5,14,15]. While circulating memory T cells maintain stable expression of secondary lymphoid organs homing receptors, such as CD62L, effector T cells lack such receptors but express peripheral tissue homing molecules [16,17]. Tissue-associated pools can be further distinguished by stable expression of CD69, where the lack of KLRG1 is characteristic of non-circulating subsets [18,19].

We have recently identified a population of CD4^+^ T cells in the livers of immune mice [5]. Considering that the priming of CD4^+^ T cells by dendritic cells takes place in secondary lymphoid organs, a likely explanation for the seeming absence of *Salmonella*-specific CD4 T cells from the spleen and the blood is that they only circulate for a limited period before moving to the liver. To test this, the current study used adoptive transfer protocols combined with antibody-mediated depletion/neutralization assays in lymphocyte deficient (*Rag2*^*-/-*^ *Il2rg*^*-/-*^) B6 mice [20] to identify protective, circulating immunity after systemic *Salmonella* infection. Moreover, a transcriptional IFN-γ reporter system [21] was exploited to study the development of protective Th1 immunity in *S*. Typhimurium immune mice. The results revealed that adoptive transfer of CD4^+^ T cells present in splenocyte pools could confer protection, but only transiently. Circulating effector Th1 cells expressed high levels of the liver-homing chemokine receptor CXCR6, suggesting that a subpopulation of protective T cells egress to peripheral tissues as immune memory develops. *Ex vivo* re-stimulation of liver memory T cells with a pool of *Salmonella* peptides, after inhibition of P2rx7/ARTC2 pathway, showed a stronger cognate response, suggesting that cell functionality was linked with tissue association, contrasting with lower reactivity of the circulating memory pool.

## Results and discussion

### Circulating immunity to *Salmonella* is seen early after vaccination but wanes with memory formation

Previous studies have shown that C57BL/6 (B6) mice vaccinated with a live, *aroA* growth-attenuated mutant strain of *S*. Typhimurium SL1344 called BRD509 clear the infection in 6–8 weeks and develop protection against a challenge infection with wild type SL1344, 10–14 weeks later [4,22]. These studies are recapitulated in S1A and S1B Fig. Antibody-mediated depletion experiments have shown that this acquired immunity is primarily T cell-mediated [4,5,23] inferring that memory should be conferred using adoptive transfer of splenocytes from immune animals. Despite an early observation in BALB/c mice to the contrary [10], adoptive transfer of memory splenocytes (10–12 weeks) in B6 mice does not confer protection to a level that is greater than that observed in mice adoptively transferred with naïve splenocytes (S1C Fig), even if the recipients are provided immune serum. To explore this phenomenon further, the splenocytes of B6 mice immunized with BRD509 for various periods were adoptively transferred to naïve recipients. We observed a degree of immunity by transfer of splenocytes 2 weeks post-vaccination (S1C and S1D Fig). Our recapitulation of previous studies on BRD509-immunized B6 mice thus demonstrated that a pool of effector cells is generated by immunization, but this pool was absent from the spleen after an early period of likely recirculation.

**Fig 1:**
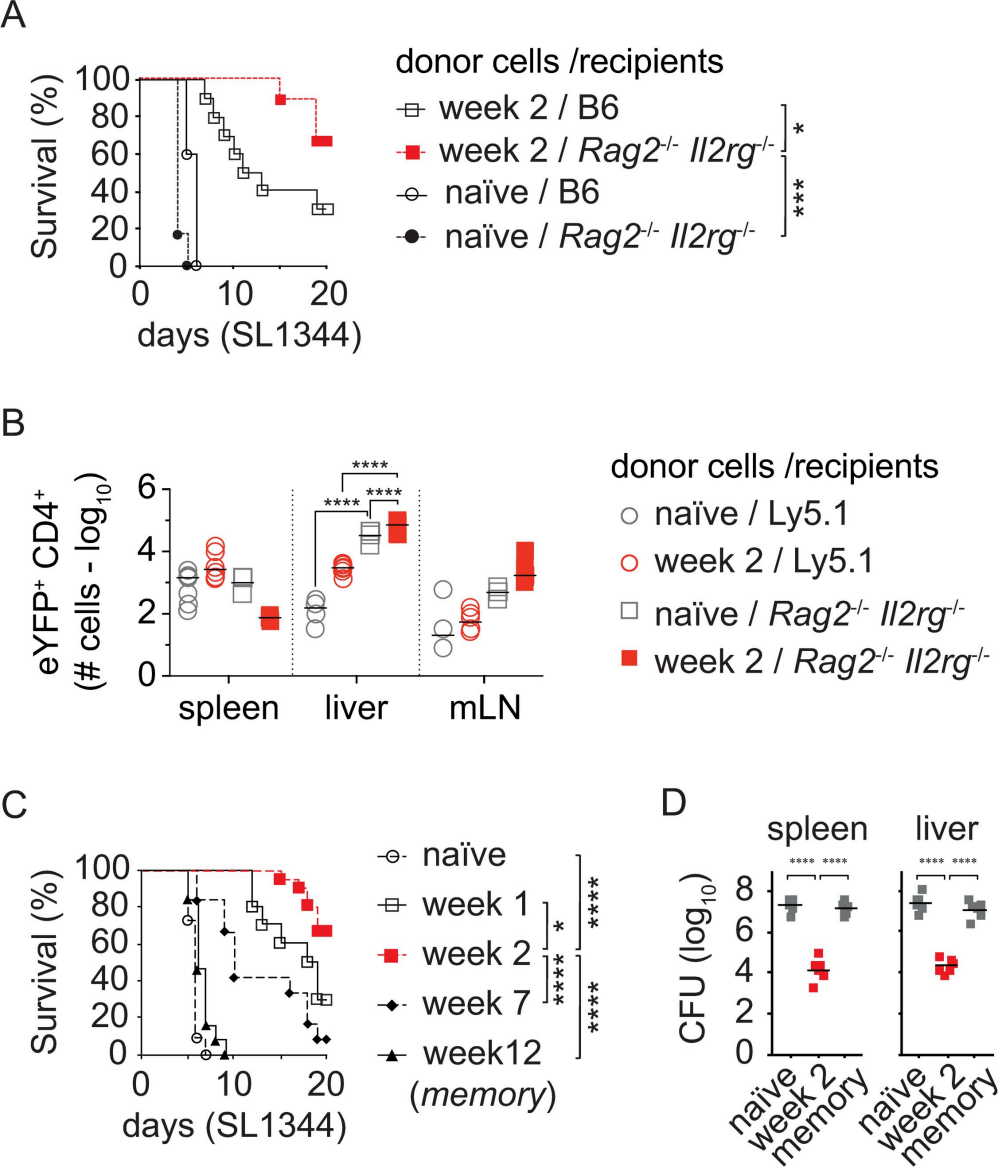
Immunity is present in the spleen at effector but not at memory phase. B6 mice (6–8 weeks old) that were either naïve or infected with 200CFU TAS2010 i.v. were culled at indicated time point p.i. as the ‘donor’ mice; where 5×10^7^ splenocytes were injected i.v. into naïve B6 or *Rag2*^*-/-*^ *Il2rg*^*-/-*^ recipient mice, which were challenged with 200CFU SL1344 i.v. 24 h later. (A) Survival of B6 or *Rag2*^*-/-*^ *Il2rg*^*-/-*^ recipient mice (n = 6–11) after receiving 5×10^7^ splenocytes from naïve or week 2 p.i. B6 donor mice, then challenged with wild type SL1344. (B) Accumulation of eYFP^+^ CD4^+^ T cells in tissues of B6 or *Rag2*^*-/-*^ *Il2rg*^*-/-*^ recipient mice (n = 3–6) 24 h after receiving 5×10^7^ splenocytes from naïve or week 2 p.i. B6 donors. (C) Survival of *Rag2*^*-/-*^ *Il2rg*^*-/-*^ recipient mice (n = 10–12) after receiving 5×10^7^ splenocytes from naïve or week 1, 2, 7 and 12 (memory) p.i. B6 donor mice, then challenged with wild type SL1344. (D) *Rag2*^*-/-*^ *Il2rg*^*-/-*^ recipient mice (n = 5–6) received 5×10^7^ splenocytes from naïve, week 2 or 12 (memory) p.i. B6 donors, then challenged with SL1344 24 h later. Shown are the bacterial loads for SL1344 in tissues of recipient mice 5 days post-challenge. For each panel, data is pooled from 2–4 independent experiments. For survival data (A, C), symbols represent percentage of surviving mice over time, and Mantel-Cox log-rank test was used for statistical analysis. For cell (B) and bacterial (D) counts, symbols represent individual mouse and lines represent geometric mean of the group, and two-way ANOVA with Bonferroni post-tests was used for statistical analysis, *, *p*<0.05; ***, *p*<0.001; ****, *p*<0.0001.

To explore this phenomenon further, we undertook a systematic analysis of immunity in *S*. Typhimurium-vaccinated animals, using a more effective vaccine designated TAS2010. This vaccine strain carries mutations in both the Entner-Doudoroff (Δ*edd*) and Embden-Meyerhof-Parnas metabolic pathways (Δ*pfkA* Δ*pfkB*) and elicits heightened protection from subsequent infection with the wild type strain SL1344 [4]. While protection can be transferred into wild type mice (Fig 1A), we reasoned that the effects were more pronounced and thus more conducive to dissection upon transfer of the cells into *Rag2*^*-/-*^ *Il2rg*^*-/-*^ recipients (which lack endogenous T cells, B cells and NK cells [20]). This was indeed the case as demonstrated by transfer of 5×10^7^ splenocytes into *Rag2*^*-/-*^ *Il2rg*^*-/-*^ or wild type B6 recipients resulting in enhanced protection compared to wild type mice (Fig 1A) and increased numbers of responding CD4^+^ T cells in the liver revealed by use of transferred splenocytes from IFN-γ reporter mouse (Fig 1B) [5,21]. This enhanced protection of *Rag2*^*-/-*^ *Il2rg*^*-/-*^ recipients is likely related to improved survival of the transferred cells in the lymphopenic environment within the first 24hr post-transfer (Fig 1B), with further accentuation by homeostatic proliferation. Though similar findings were obtained from experiments with splenocytes from BRD509-immunized mice (S1E Fig); the overall survival rates in recipient mice reflected the respective immunogenicity of the vaccine strains where TAS2010 induces enhanced protection [4]. Moreover, while adoptively transferrable protection was seen early after TAS2010 vaccination, neither splenocytes from 12-week TAS2010-immunized mice nor from naïve mice conferred protection to *Rag2*^*-/-*^ *Il2rg*^*-/-*^ mice (Fig 1C). The evidence of circulating cellular correlates of immunity before the memory stage was supported by an analysis of the bacterial counts which revealed low SL1344 counts in both the spleen and liver of mice that received the splenocytes of mice vaccinated for 2 weeks with TAS2010 (Fig 1D). In two different sets of control experiments (detailed in the S2 Fig), we demonstrated that week 2 splenocyte-mediated protection is: (I) directly proportional to the number of cells transferred (S2A Fig) and (II) not driven by any carry-over of the vaccine strain that was transferred along with immune cells (S2B Fig). Of note, no TAS2010 were recovered from the organs of recipient mice challenged with SL1344. These studies suggested that live-attenuated *Salmonella* strains can induce highly protective cellular immunity in the spleen that gradually wanes in its ‘transferability’ as the vaccine is cleared, and T cell memory is formed.

## Effector T helper 1 immunity is essential for adoptively transferred protection against lethal *Salmonella* infection

**Fig 2:**
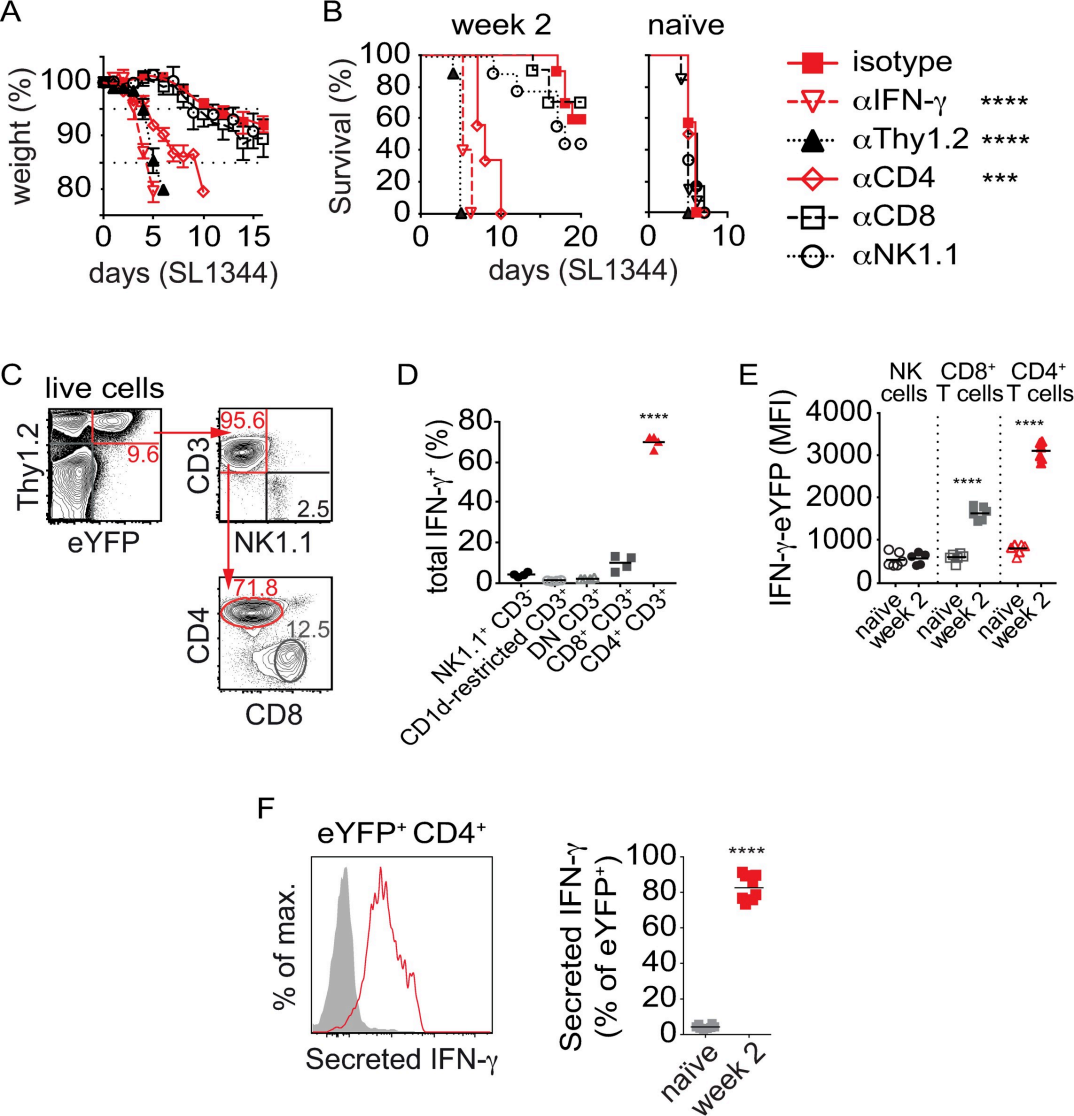
Adoptive protection from week 2 splenocytes is IFN-γ and CD4^+^ T cell-dependent. B6 or IFN-γ-eYFP^in/in^ mice were either naïve or infected with 200CFU TAS2010 i.v. for 2 weeks. (A) Weight loss (expressed as a percentage of initial weight, group mean shown) and (B) percentage survival of *Rag2*^*-/-*^ *Il2rg*^*-/-*^ recipient mice after receiving 5×10^7^ splenocytes from naïve or week 2 p.i. B6 donor mice, i.p. injected with either isotype control, anti-Thy1.2, anti-CD4, anti-CD8 or anti-NK1.1 i.p. at the time of cell transfer, then challenged with 200CFU wild type SL1344 24 h after transfer (n = 9–10). Data are pooled from 2–4 independent experiments. Within week 2 p.i. donor splenocytes, (C) representative FACS plots and (D) percentage of the expression of IFN-γ-eYFP in different Thy1.2^+^ sub-populations and E) level of IFN-γ expression (expressed as MFI) identify CD4^+^ T cells are the main producer of IFN-γ. Each symbol represents a pool of donor cells from an independent experiment, data from 4–7 experiments are shown here with horizontal bar representing the mean for each sub-population, and one- or two-way ANOVA with Bonferroni post-tests was used for statistical analysis. F) Representative FACS histogram and graph show the majority of eYFP^+^ CD4^+^ T cells in week 2 p.i. mice secreted IFN-γ upon *ex vivo* restimulation with heat-killed TAS2010. Symbols in the graph represent data from individual animals (n = 8 per each group) with the group mean shown as horizontal bars. For survival data (B), The Mantel-Cox log-rank test was used for statistical analysis. For all other panels, one- two-way ANOVA with Bonferroni post-tests was used for statistical analysis, *, *p*<0.05; ***, *p*<0.001; ****, *p*<0.0001.

To identify the cells conferring such protection in the splenic pool, adoptive splenocyte transfers into *Rag2*^*-/-*^ *Il2rg*^*-/-*^ recipients were combined with *in vivo* antibody-mediated cell depletion/neutralization of donor lymphoid subsets and cytokine [5]. Depletion of Thy1.2^+^ splenocytes or neutralization of IFN-γ in *Rag2*^*-/-*^ *Il2rg*^*-/-*^ recipients led to rapid development of disease symptoms as reflected by weight loss in SL1344-challenged mice (Fig 2A). Depletion of cells expressing CD4, but neither CD8 nor NK1.1, significantly reduced adoptively transferred protection in *Rag2*^*-/-*^ *Il2rg*^*-/-*^ recipients, as measured by weight loss (Fig 2A) and survival post-challenge (Fig 2B). SL1344-challenged recipients treated with anti-IFN-γ, anti-Thy1.2 or anti-CD4 mAbs became moribund within 10 days post-challenge (Fig 2B). A similar reduction in protection was observed upon transfer of 5 × 10^7^ spleen cells from vaccinated CD4^+^ T cell-deficient (MHC-II^-/-^) donors. Depletion following antibody-treatment did not alter the already poor survival of mice receiving naïve splenocytes (Fig 2B). After two weeks of infection with TAS2010, eYFP expression, a proxy for IFN-γ production, was observed exclusively in Thy1.2^+^ subsets (Fig 2C); and 70% of IFN-γ^+^ cells were CD4^+^ T cells (Fig 2D). Th1 cells displayed the highest expression of the IFN-γ reporter activity among the detectable groups, as measured by median fluorescence intensity (MFI) (Fig 2E). Upon *ex vivo* re-stimulation with TAS2010-lysate, 82% of eYFP^+^ CD4^+^ T cells secreted IFN-γ, whereas Th1 cells from naïve mice did not respond to the same stimuli (Fig 2F). This experiment confirmed that week 2 CD4^+^ T cells in the spleen recognized *Salmonella* antigens. Intriguingly, a series of tests attempting to protect *Rag2*^*-/-*^ *Il2rg*^*-/-*^ or wild type B6 recipients with transfer of purified Th1 cells from week 2 splenocytes failed, indicating that circulating Th1 cells were essential but not sufficient for adoptively-transferred protection. This observation suggests that CD4^+^ T cells participate in, or coordinate the function of, a cellular network that collectively provides protection against secondary infection.

### Anti-*Salmonella* CD4^+^ T cells express CXCR6 chemokine receptor and accumulate in the liver as the vaccine strain is cleared

**Fig 3:**
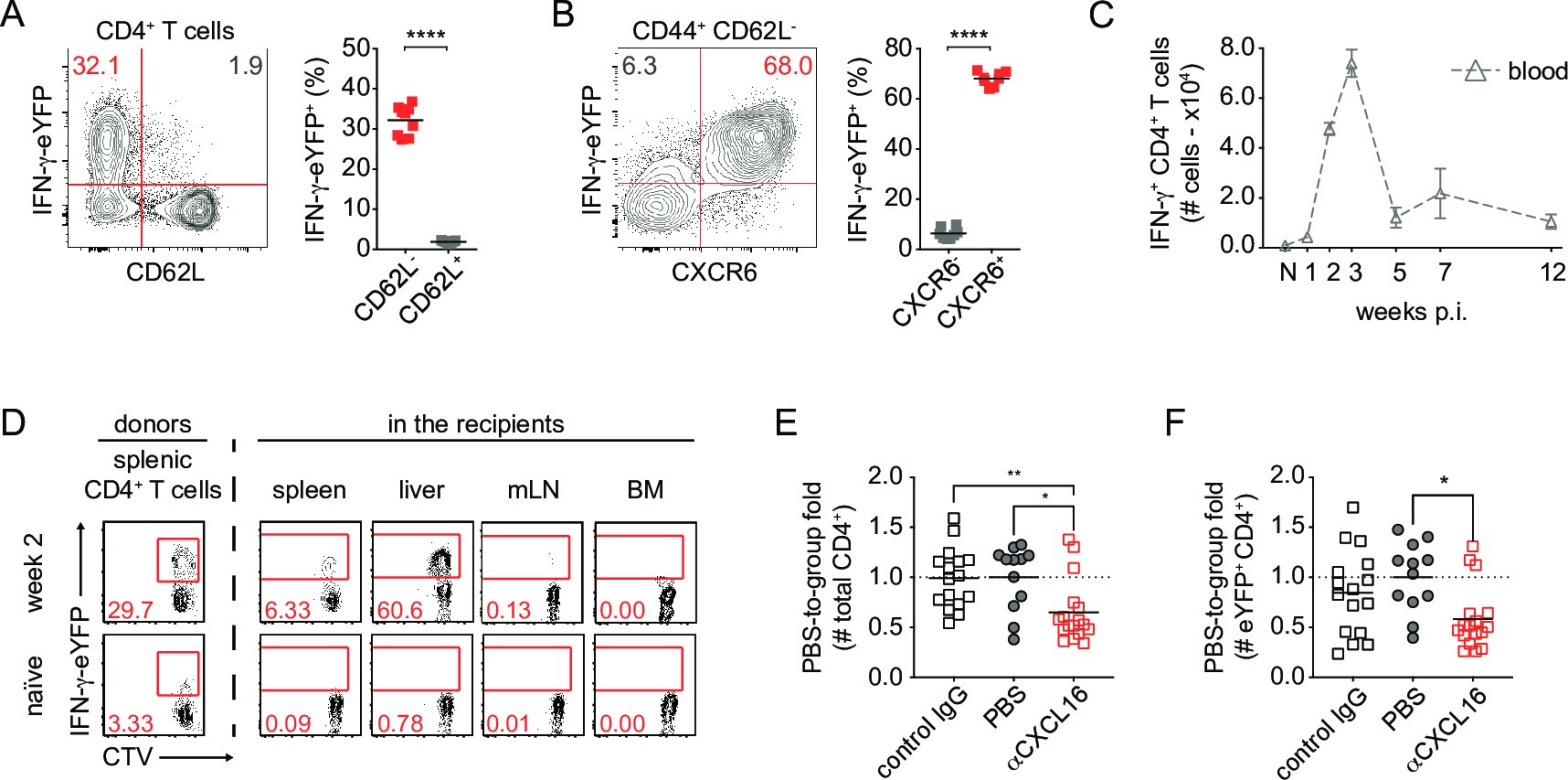
Circulating Th1 cells home to the liver as primary *Salmonella* infection is resolved. IFN-γ-eYFP^in/in^ mice were infected with 200 CFU TAS2010 i.v. and 2 weeks later splenocytes were harvested. (A) Representative FACS plot, and graph showing IFN-γ-eYFP is predominately expressed within the CD62L^-^ subpopulation of CD4^+^ T cells in the spleen (n = 7–8). (B) Representative FACS plot, and graph showing that within the CD44^+^CD62L^-^ sub-population of CD4^+^ T cells, IFN-γ-eYFP is co-expressed with CXCR6 (n = 7–8). (C) Absolute numbers of IFN-γ-eYFP^+^ CD4^+^ T cells in 100 μL blood over time after infection with 200CFU TAS2010 i.v. (n = 4–12). (D) Donor splenocytes from naïve or week 2 p.i. IFN-γ-eYFP^in/in^ mice were labelled with CellTrace Violet (CTV) and adoptively transferred into *Rag2*^*-/-*^ *Il2rg*^*-/-*^ recipient at 5×10^7^ cells/mouse. Representative FACS plots show IFN-γ-eYFP and CTV level within CD4^+^ T cells accumulated in different tissues, 24 h post-transfer. Liver uptake of transferred (E) total CD4^+^ T cells and (F) eYFP^+^ CD4^+^ T cells was measured 24 h post-transfer in *Rag2*^*-/-*^ *Il2rg*^*-/-*^ recipient mice (n = 12–17) that were pre-treated with αCXCL16 neutralizing antibodies, control IgG or PBS 24hr prior to cell transfer. Data is expressed as fold-change against the PBS group mean. For each panel, data is pooled from 2–4 independent experiments. For statistical analyses, student *t*-test, one- or two-way ANOVA with Bonferroni post-tests were used, *, *p*<0.05; ***, *p*<0.001; ****, *p*<0.0001.

While there is no licensed vaccine against human invasive non-typhoidal salmonellosis (iNTS) infection, there has been some analysis of T cell responses after vaccination with *S*. *enterica* var Typhi, a related pathogen in humans. Human volunteer studies using immunization with live CVD909 or Ty21a vaccines identified *Salmonella*-specific T cells in the blood of vaccinated groups that were predominantly CD62L^-^ and expressed markers of tropism for peripheral tissues [13,24]. This phenotype was identified 7–21 days post-vaccination [12] and was followed by a reduction in *Salmonella*-specific T cells in the circulation [13]. In our murine model, week 2 splenic IFN-γ^+^ CD4^+^ T cells lacked CD62L expression (Fig 3A), and CD44^hi^ IFN-γ^hi^ cells upregulated the chemokine receptor CXCR6 (Fig 3B), a receptor which is associated with T cell homing to the liver [25]. Similar expression profiles were observed in IFN-γ^hi^ CD4^+^ T cells in the blood, and a sharp decrease in cell numbers in the blood was evident from week 5 post-vaccination (Fig 3C), recapitulating observations infected with a vaccine strain [13]. To test whether CXCR6^+^ Th1 splenocytes after *S*. Typhimurium infection relocated to the periphery, week 2 spleen cells were labelled with CellTrace Violet (CTV) and transferred to uninfected *Rag2*^*-/-*^ *Il2rg*^*-/-*^ recipients (Fig 3D). At 24h post-transfer, the spleen, liver, mesenteric lymph nodes (mLN) and bone marrow (BM) were harvested and analyzed for cell proliferation. In the spleen, 30% of total CD4^+^ T cells from infected mice were transcriptionally active for IFN-γ as determined by eYFP expression, in contrast with naïve animals, where the Th1 cells represented only 3.3% of CD4^+^ T cells (Fig 3D). One day after transfer, effector Th1 splenocytes redistributed to the liver of the recipients, where 60% of the transferred CD4^+^ T cells were eYFP^+^ (Fig 3D). CXCL16 is the only known ligand for CXCR6 [26] and its activity can be blocked using neutralizing antibodies (S3 Fig). We injected these antibodies against CXCL16 24 h before adoptive transfer of week 2 splenocytes and observed a significant reduction in the accumulation of CD4^+^ T cells in the recipient liver 24 h later (Fig 3E). A similar magnitude of decrease in eYFP^+^ CD4^+^ T cell counts was observed in mice where CXCL16 was neutralized, however the fold-change observed between the control IgG- and anti-CXCL16-treated groups was not statistically significant in 3 pooled experiments (Fig 3F). In considering previous reports where CXCR3 and CCR5 [25,27] compensated for some effects of CXCR6 deficiency in Th1 cells, and that the impact of CXCR6 on liver homing by CD8^+^ T cells can change over time [28–30], we conclude that the role of CXCR6 in liver immunity to *Salmonella* infection is complex and as such requires further investigation to fully explain the role of the chemokine and its ligand in cell accumulation in this organ.

**Fig 4:**
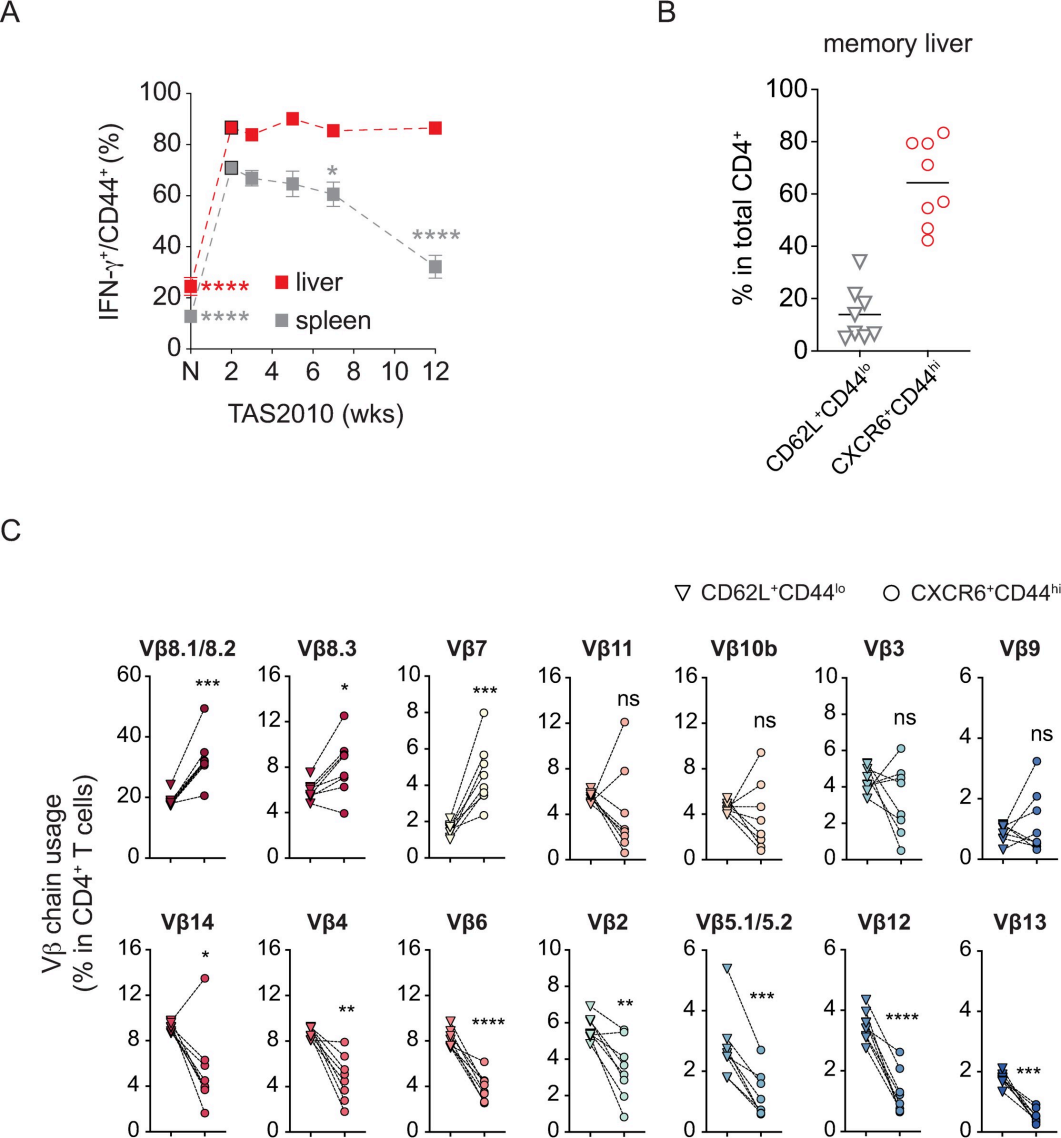
Liver migratory CD4^+^ T cells show preferential usage of selected Vβ chains compared to naïve cells. IFN-γ-eYFP^in/in^ or B6 mice were i.v. infected with 200CFU TAS2010. At week different time points p.i. mice were euthanased; the liver was then perfused with PBS to remove circulating cells. (A) Percentage of IFN-γ-eYFP^+^ cells among CD44^+^ CD4^+^ T cells in the spleen and liver (n = 4–12). (B) The majority of liver CD4^+^ memory (week 12) T cells displayed a CXCR6^+^CD44^hi^ phenotype, whereas CD62L^+^CD44^lo^ naïve cells were present in lower numbers. (C) The frequency of 15 individual Vβ chains within total or indicated subset of liver CD4^+^ memory T cells were assessed for individual. Together these chains accounted for 78% (range: 74.4–85.5%) of total CD4^+^ T cells. For each individual Vβ chain, its frequency in the CXCR6^+^CD44^hi^ CD4^+^ T cells was compared to that in the naïve subset in the same animal; for better visualisation, a dotted line is drawn between matching data points from the same individual (n = 8). (A) Scares represent mean and bars represent SEM. (B and C) Triangles represent CXCR6^+^CD44^hi^ cells, and circles represent CD62L^+^CD44^lo^ cells. Paired *t-*test was used to determine whether the frequency was significantly altered between the two subsets: *, *p*<0.05; **, *p*<0.01; ***, *p*<0.001; ****, *p*<0.0001; ns, not significant.

CD4^+^ T cells can produce IFN-γ in response to TCR stimulation, but antigen-experienced CD4^+^ T cells with irrelevant specificities can also secrete IFN-γ in a non-cognate manner [31]. To determine if at least some of the CD4^+^ T cells accumulating in the liver were specific for the infection, we re-examined our data on antigen-experienced (CD44^hi^) CD4^+^ T cells, a population that should include those cells responding to the *Salmonella* antigens in the spleen and liver. Fig 4A shows that CD4^+^ T cells in both organs rapidly gained the ability to produce IFN-γ. Interestingly, the proportion of IFN-γ^+^ cells amongst CD44^hi^ CD4^+^ T cells declined in the spleen over time (Fig 4A), while IFN-γ-expressing cells dominated the CD4^+^ T cell population in the liver. Even at the point where memory was well established, evidenced by protection against virulent challenge (i.e. at week 12, ref [4]), more than 80% of CD44^hi^ CD4^+^ T cells remained eYFP^+^ in the liver (Fig 4A). To determine the clonality of this population in the liver of *Salmonella*-infected mice, we analyzed the variable region of the TCR β chain (Vβ) of IFN-γ-eYFP^+^ memory CD4^+^ T cells, and compared Vβ usage to that of non-activated (i.e. naïve T cells) from the same animals to exclude animal to animal repertoire bias. A gating strategy was designed to estimate the usage of Vβ chains by CD44^hi^ CD4^+^ T cells, as a window into TCR selection by the infection. CD44, CD62L and CXCR6 expression were used to analyze CD4^+^ T subsets in the liver where CD62L^+^CD44^lo^, and CXCR6^+^CD44^hi^ subsets, were defined as naïve CD4^+^ T cells and memory Th1 cells, respectively (S4A Fig). On average, naïve cells comprised 14% of the total CD4^+^ T cells found in the liver of mice that had cleared infection; whereas 64% of the recovered CD4^+^ T cells assumed a memory phenotype (Fig 4B). The frequencies by which naïve and memory cells used specific Vβ chains in the same individual were analyzed (Fig 4C). Overall, the distributions of specific TCR Vβ chains vary among mice vaccinated with *S*. Typhimurium supporting previous studies showing limited clonality of CD4^+^ T cell responses to pathogens [32,33]. Vβ8.1/8.2, Vβ8.3 and Vβ7 chains showed enrichment, whereas Vβ14, Vβ4, Vβ6, Vβ2, Vβ5.1/5.2, Vβ12 and Vβ13 decreased in relative abundance in Th1 memory cells compared with naïve cells (Fig 4C). These observations indicate that there is a level of selection bias, i.e. specificity, that occurs in T cells present in the liver during and after clearance of the live vaccine strain. While non-cognate contributions towards IFN-γ provision are still possible, these finding suggest that the majority of antigen-experienced cells, tracked via their enhanced IFN-γ-eYFP expression, include cells that were primed against *Salmonella* antigens.

### P2rx7 pathway blockade is needed to identify *Salmonella*-specific memory CD4^+^ T cells that have relocated to the immune liver

**Fig 5:**
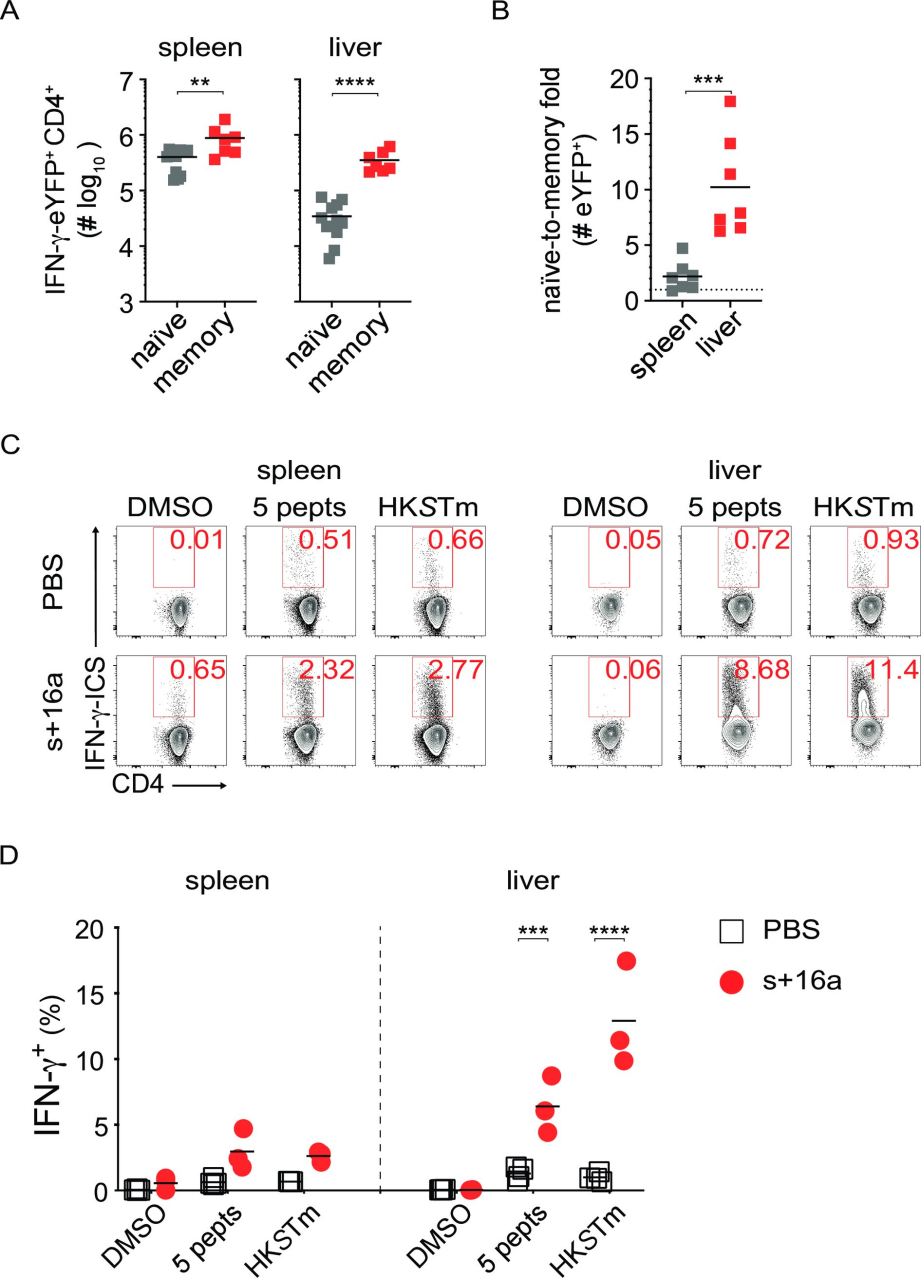
Specific blockade of ARTC2 preserve functional activity in *Salmonella*-specific CD4^+^ T cells in the liver. IFN-γ-eYFP^in/in^ or B6 mice were either naïve or i.v. infected with 200CFU TAS2010. At week 12 p.i., 50μg of s+16a nanobody was injected 15-20min immediately prior to euthanasia; the liver was then perfused with PBS to remove circulating cells. (A) The number of IFN-γ-eYFP-expressing CD4^+^ T cells is shown for the spleen and liver from naïve or memory mice (n = 7–11). (B) The number of IFN-γ-eYFP-expressing CD4^+^ T cells is expressed as fold-change memory/naïve in organs (n = 7–11). (C and D) To analyze CD4^+^ T cells with or without s+16a nanobody, naïve or memory B6 animals were injected i.v. with either 50μg of s+16a nanobody or PBS 15min prior to organ harvest. Total splenocytes and liver leucocytes were re-stimulated *ex vivo* with of either a pool of 5 *Salmonella* peptides (5 pepts, 1μg each/well) or heat-killed SL1344 (HK*S*Tm, 5×10^7^cfu/well), or DMSO (negative control). After 14h post-exposure to cognate stimuli, 10 μg/ml brefeldin A was added for a further 4 h to stop cytokine secretion. Cells were then prepared for surface and intracellular staining. Shown are (C) representative FACS plots and (D) percentages of IFN-γ^+^ among CD4^+^ T cells (n = 3–5). Data is representative of 3 independent experiments. Student *t-*student tests (or with adjustment for multiple testing) were used to statistical analyses, **, *p*<0.01; ***, *p*<0.001; ****, *p*<0.0001.

With clearance of the growth-attenuated *Salmonella* strain, both the memory spleen and liver harbor larger numbers of IFN-γ-eYFP^+^ CD4^+^ T cells than age-matched uninfected mice (Fig 5A). Livers from infected mice had a 10.2-fold increase in Th1 cell numbers than the respective naïve compartments (Fig 5B). Together with the preceding data, this finding suggests that IFN-γ^+^ CD4^+^ T cells relocate from the blood into the liver. Isolation of T cells associated with tissues, such as liver, is made difficult by a curious phenomenon whereby the isolation procedure activates ARTC2 [5]. ARTC2 subsequently ribosylates P2xr7 and the resulting potassium influx results in the death of many isolated cells during processing for ex vivo analysis [34,35]. This isolation-associated phenomenon can be prevented by specific blockade of ARTC2 with s+16a nanobody [5]. To assess whether blockade of ARTC2 could indeed reveal memory Th1 cells that immigrated into the liver from the blood, 12 week-infected B6 mice were injected with 50μg s+16a or PBS in the tail vein 15 min before euthanasia [5,36]. Liver leucocytes and splenocytes were stimulated *ex vivo* with 5×10^7^ CFU heat-killed *S*. Typhimurium (HK*S*Tm), or a pool of five *Salmonella*-peptides that were identified in previous studies as CD4^+^ T cell antigens (FliC_429-443_, GroEL_40-53_, LpdA_338-351_, SseI_268-280_ and SseJ_329-341,_ 5 pepts) [32,33,37,38]. While we could only recover small fractions of *Salmonella*-responsive CD4^+^ T cells from control mice, CD4^+^ T cells from mice treated with s+16a or PBS had significantly increased IFN-γ production upon *ex vivo* re-stimulation with either 5 pepts or HK*S*Tm (Fig 5C and 5D). Interestingly, this enhanced recovery of IFN-γ^+^ CD4^+^ T cells only occurred when the mice were treated with the s+16a *in vivo*, but not by exposing CD4^+^ T cells from immune mice to s+16a *in vitro* after isolation (S5A and S5B Fig). Combined with observations that there was no difference in the *ex vivo* MFI of eYFP in CD69^+^ and CD69^-^ sub-populations of eYFP^+^ CD4^+^ T cells (S5C Fig), these findings argue against the nanobody enhancing IFN-γ production [35] and instead support the conclusion that ARTC2/P2rx7 inhibition assisted in identifying true memory Th1 cells in the context of the liver, by aiding their survival.

In conclusion, our studies argue that, upon immunization by effective live attenuated *Salmonella* vaccines, antigen-specific CD4^+^ T cells differentiate into Th1 cells with protective potential that circulate for a short period of time before eventually locating to the liver, in a manner that is at least in parts dependent on the CXCL16/CXCR6 axis. These findings resolve some of the puzzling observations regarding transferable immunity from *Salmonella* infection or vaccination and provide new insights into the spatiotemporal characteristics that underpin localized protection in the liver as we have previously demonstrated [5]. The study reported here reveals that the spleen of an immunized mouse harbors immunity only transiently, clears the local infection, with little apparent memory preserved *in situ*. The role of the pathogen in the relocation of immunity to the periphery, perhaps via inducing chemokine upregulation in infected cells, warrants further investigation.

## Materials and methods

### Ethics statement

Animal experiments were approved by the University of Melbourne Animal Ethics Committee (AEC) and conducted according to National Health and Medical Research Council (NHMRC) Australian Code of Practice for the Care and Use of Animals for Scientific Purposes (2013).

### Bacteria. *S*. Typhimurium

Antibiotic resistant *S*. Typhimurium strains were inoculated into LB broth supplemented with 50 μg/ml streptomycin and grown shaking at 180 rpm, at 37°C overnight. For use in experiments, 100 μl of the overnight culture was subcultured into fresh LB broth supplemented with 50 μg/ml streptomycin at 37°C for 3-4h for SL1344 and Δ*edd* Δ*pfkA* Δ*pfkB* mutant (TAS2010) and 5h for Δ*aroA* mutant (BRD509), until an OD_600_ between 0.6–0.8 reading was reached. Glycerol (80% v/v) was added to the broth at a 1:10 ratio, and subsequently 500 μl aliquots were stored at -80°C until required. Aliquots were safe for estimation of purity, via metabolic and PCR tests [4], and bacterial counts.

### Mice and infection models

Age- and sex-matched C57BL/6 (B6), *Ptprc*^*b*^ (CD45.1^+/+^—Ly5.1) B6, *Rag2*^*-/-*^ *Il2rg*^*-/-*^ B6 [20], IFN-γ-eYFP^in/in^ B6 [21] and *IAE*^*-/-*^ B6 [39] mice were bred under specific pathogen free (SPF) conditions at the Biological Research Facility (BRF), at the Peter Doherty Institute for Infection and Immunity. IFN-γ-eYFP^in/in^ B6 mouse was generously provided by Prof. R. Locksley, University of California, San Francisco, USA [21]. Mice received 200 CFU of *S*. Typhimurium in 200 μl PBS via intravenous (i.v.) injection.

### Estimation of survival

Euthanasia was performed whenever mice challenged with SL1344 dropped to less than 85% from the initial body weight and presented signs of distress, as approved by the AEC.

### Bacterial counts

Spleen and liver single-cell suspensions were prepared by pushing these organs through 70 μm Nylon Cell Strainers (BD Falcon) using syringe plunges and sterile PBS. To enumerate the bacteria, dilutions of 100 μl of the single cell suspension of the organs were plated on solid LB or XLD media supplemented with 50 μg/ml of streptomycin at 37°C overnight. Blood was collected from euthanized mice by cardiac puncture with a 25G needle and immediately transferred to a heparinized blood collection tube (BD Vacutainer). Dilutions of 100 μl of blood were cultured on LB agar plates supplemented with 50 μg/ml of streptomycin at 37°C overnight to enumerate the bacteria.

### Lymphocyte isolation and enrichment

The spleen or mLN was gently pushed through a 70 μm nylon cell strainer (BD Falcon) to make a single-cell suspension. After centrifugation (525 ×g, 5 min, 4°C), splenocytes were resuspended and incubated in red blood cell lysis TAC buffer (17 mM Tris and 140 mM ammonium chloride, pH 7.2) for 10 min, rotating at RT, to lyse the red blood cells. Liver was perfused via hepatic portal vein immediately after euthanasia and then gently pushed through a 70μm Nylon Cell Strainer (BD Falcon). After centrifugation (525 ×g, 5 min, 4°C), liver cells were resuspended and incubated in TAC buffer for 10 min, rotating at RT. After centrifugation, liver leucocytes were enriched using 35% isotonic Percoll and centrifuged at 930 ×*g* for 12 min, without deceleration, at RT. The two top layers were carefully removed using disposable pipettes. Blood was collected via cardiac puncture into heparinized blood collection tube (BD Vacutainer) and resuspended in TAC buffer for 10 min at RT. Left femur was collected and with the help of 25G needle the bone marrow (BM) was plunged with an ice-cold RPMI-1640 + 2% (v/v) FCS solution. After centrifugation, BM was resuspended and incubated in red blood cell lysis TAC buffer, followed by wash step. Single-cell suspensions were resuspended in ice-cold RPMI-1640 + 2% (v/v) FCS + 1mM EDTA until use.

### Staining and flow cytometry

#### Surface staining

One million cells from the spleen, liver, mLN, BM, or blood were blocked with α-mouse CD16/CD32 Fc block (2.4G2, BD Biosciences) in 50 μl FACS buffer (0.1% (w/v) BSA, 5mM EDTA in sterile PBS) for 15 min on ice. Surface markers were stained in 50 μl of FACS buffer using antibodies and tetramers listed in S1 Table, for 30 min on ice and in the dark. Stained cells were washed three times with FACS buffer and strained with either propidium iodide (PI) at 1 μg/10^6^ cells and approximately 2 × 10^4^ blank calibration beads (SPHERO_,_ 6.0–6.4 μm, BD Biosciences) for dead cell exclusion and estimation of absolute cell number, respectively. ***Intracellular staining (ICS)*.** Single-cell suspensions stained for surface markers and Fixable Viability Dye eFluor 780 (eBioscience) were fixed and permeabilized using the eBioscience FoxP3 Fixation/Permeabilization kit, according to manufacturer’s instructions. Intracellular antigens were stained in 50 μl permeabilization buffer for 30 min at 4°C. ***Analysis*.** BD LSR II and BD LSRFortessa Cell Analysers were used for collection of flow cytometric data, which was analyzed post-recording using the FlowJo software v10.

### Labelling with proliferation dyes

Single splenocyte suspensions were prepared as mentioned above and stained following manufacturer’s recommendation. Briefly, cells were pelleted and resuspended in 1 ml pre-warmed (37°C) FACS buffer (PBS + 0.1% BSA) per 1 × 10^7^ cells. 1 μl of CellTrace Violet dyes (ThermoFisher) was added per 1 × 10^6^ cells and immediately vortexed for homogeneous staining. Cells were incubated in a 37°C water bath for 10 min and the reaction was stopped with two wash steps in ice-cold RPMI-1640 + 10% FCS.

### Adoptive transfer of lymphocytes

Single splenocyte suspensions were prepared as described above, pooled from different donor mice and incubated in RPMI-1640 supplemented with 5% (v/v) FCS and 100 μg/mL gentamycin for 20 min at 37°C. Splenocytes were washed twice in ice-cold PBS and then counted. Cell numbers were estimated using a hemocytometer, where 0.4% trypan blue was used for dead cell staining. Viable splenocytes were washed twice in ice-cold PBS, diluted to 5 × 10^7^ splenocytes per 200 μl in PBS, and injected into recipients via the tail vein.

### *Ex vivo* stimulation of CD4^+^ T cells

Previously reported *Salmonella* MHC-II peptides (FliC_429-443_, GroEL_40-53_, LpdA_338-351_, SseI_268-280_ and SseJ_329-341_) were synthesized to >90% purity (GL Biochem). For preparation of heat-killed *Salmonella* (HKSTm), *S*. Typhimurium SL1344 was grown statically in Luria broth with 50 μg/ml streptomycin at 37°C overnight. The overnight culture was washed twice in sterile PBS, resuspended in PBS and heat-killed at 60°C for 60 min, then stored at -20°C until use. For *ex vivo* re-stimulation, cells were seeded in round-bottom 96-well plates at 2×10^6^ cells per well in RPMI10 (10% FCS, 2mM L-glutamax, 100μg/ml streptomycin, 100 U/ml penicillin), and to each well 1μg peptide and 5×10^7^cfu HKSTm was added to a total of 200μl/well. Cells were incubated for 18–20 hours at 37°C with 5% CO_2_, with GolgiPlug (BD Bioscience) added in the final 4 hours of incubation.

### *In vivo* antibody-mediated depletion of lymphocytes or neutralisation of IFN-γ and CXCL16

*In vivo* antibody depletion/neutralization was performed by i.p. injections of monoclonal antibodies (mAb), using doses and frequencies as listed in S2 Table, and as previously indicated [4].

### Nanobody-mediated blockade of P2rx7/ARTC2 pathway

Mice were injected i.v. with 50 μg of s+16a nanobody (BioLegend), diluted in 200 μl PBS, 15 min prior to euthanasia and organ harvest, as protocoled elsewhere [40].

## Supporting information

S1 Fig Adoptive transfer of BRD509-immune splenocytes fails to protect cell recipients from lethal challenge. B6 mice (6–8 weeks old) were infected i.v. with 200CFU BRD509 strain. (A) Bacteria counts on solid LB media from homogenate spleen of infected mice over the course of the primary infection. (B) Survival of mice immunised with BRD509 i.v., or uninfected, and 14 weeks later challenged with 200CFU SL1344 i.v.. (C) Survival of groups of mice that adoptively received 5×10^7^ total donor splenocytes harvested at weeks 1, 2, 3, 4, 6, 14 post-single BRD509 infection and 24h post-transfer were challenged with 200CFU SL1344 iv. (D) SL1344 counts on differential, solid XLD media from spleen and liver of B6 mice recipients of week 2 or naïve donor splenocytes on day 4 post-challenge. (E) Survival of either complete (B6) of lymphocyte-deficient (*Rag2*^*-/-*^ *Il2rg*^*-/-*^) mice that adoptively received 5×10^7^ total naïve or week 2 BRD509 donor splenocytes and 24h post-transfer were challenged with 200CFU SL1344 i.v.. Data is representative of (A,B,E) 2–4 and (C,D) 2 pooled independent experiments with (A) 4–8, (B) 10, (C) 5–7, (D) 5–10 and (E) 6–11 samples per group. (A) Symbols and bars represent mean and SEM, (B,C,E) symbols represent percentage of survivors, and (D) lines and symbols represent mean and individual measurements. respectively. Statistical analysis, log-rank (Mantel-Cox), multiple t-student test. ***p<0.005, ****p<0.001.
(TIF)

S2 Fig Carryover vaccine strain does not confer protection to cell recipients against lethal challenge. (A) Weight loss (left) and survival (right) of *Rag2*^*-/-*^ *Il2rg*^*-/-*^ mice that received different numbers of splenocytes from week 2-TAS2010 infected mice, and the recipients were challenged with 200CFU SL1344 24h after adoptive transfer. (B) Weight loss (left) or survival (right) of *Rag2*^*-/-*^ *Il2rg*^*-/-*^ mice that received i.v. 5×10^7^ splenocytes from either week 2-TAS2010 infected B6, or uninfected B6, or did not receive cells; control that were adoptively transferred ~200 CFU TAS2010 along with splenocytes (*) or injected with 200CFU TAS2010 i.v. at the time of transfer. 24h post-transfer recipients were challenged with SL1344 or left unchallenged. Data is representative of (A,B) 2 pooled independent experiments, with (A) 10 (B) 7–10 animals per group. Statistical analysis, log-rank (Mantel-Cox). **p<0.01, ***p<0.005.
(TIF)

S3 Fig Neutralization of CXCL16 in vitro. (A) Neutralization of CXCL16 was measured by the inhibition of migration of murine CXCR6-expressing B300.19 cells towards soluble CXCL16 *in vitro* [26]. Symbols represent mean and bars represent SEM. Data is representative of pooled independent experiments, with 3–4 samples per group.
(TIF)

S4 Fig Gating strategy for the analysis of Vβ chain in memory and naïve CD4^+^ T cells in the liver. (A) B6 mice were immunised with 200CFU TAS2010 i.v. 12 weeks later, the mice were killed, the livers perfused with PBS and the cells collected. Representative FACS plots are also provided for gating on CXCR6^+^CD44^hi^ and CD62L^+^CD44^lo^ subsets for liver CD4^+^ T cells.
(TIF)

S5 Fig s+16a nanobody does not directly alter IFN-γ production *ex vivo*. Wild type B6 mice were either naïve or infected with 200CFU TAS2010 i.v. At week 5 p.i., splenocytes were harvested and re-stimulated ex vivo in the presence of indicated concentration of s+16a nanobody at 2×10^6^ cells per well in 200μl (n = 4). The nanobody was present during the entirety of the re-stimulation protocol, either with (A) PMA and ionomycin for 4h with brefeldin A, or (B) a pool of *Salmonella* peptides (5pept, as per Fig 5) for 18 h with brefeldin A added in the final 4 h. At the end of the incubation period, re-stimulated cells were intracellularly stained for IFN-γ. No statistically significant difference (one-way ANOVA with Bonferroni post-tests) was observed between re-stimulated cells treated with different concentration of s+16a nanobody. (C) IFN-γ-eYFP^in/in^ mice were infected with 200CFU TAS2010 i.v. for 12 weeks. Shown is the geometric mean fluorescence intensity (gMFI) of IFN-γ-eYFP in both CD69^+^ and CD69^-^ CD4^+^ T cells in mice that were pre-injected with either 50μg of s+16a nanobody or equal volume of PBS 15-20min immediately prior to euthanasia and tissue collection (n = 6–8). The liver was then perfused with PBS to remove circulating cells. No statistically significant difference (Student t-test) was observed between s+16a or PBS pre-injected mice. Symbols represent data from individual mice, mean±SEM shown.
(TIF)

S1 Table List of antibodies and tetramer for flow cytometry analysis. (TIF)

S2 Table Purified antibodies for cell and cytokine depletion. (TIF)
