# Supplementary figures and images for: CD4+ T cell immunity to Salmonella is transient in the circulation
Source: PLoS Pathog. 2021 Oct 25;17(10):e1010004. doi: 10.1371/journal.ppat.1010004 (PMC8568161; doi:10.1371/journal.ppat.1010004)

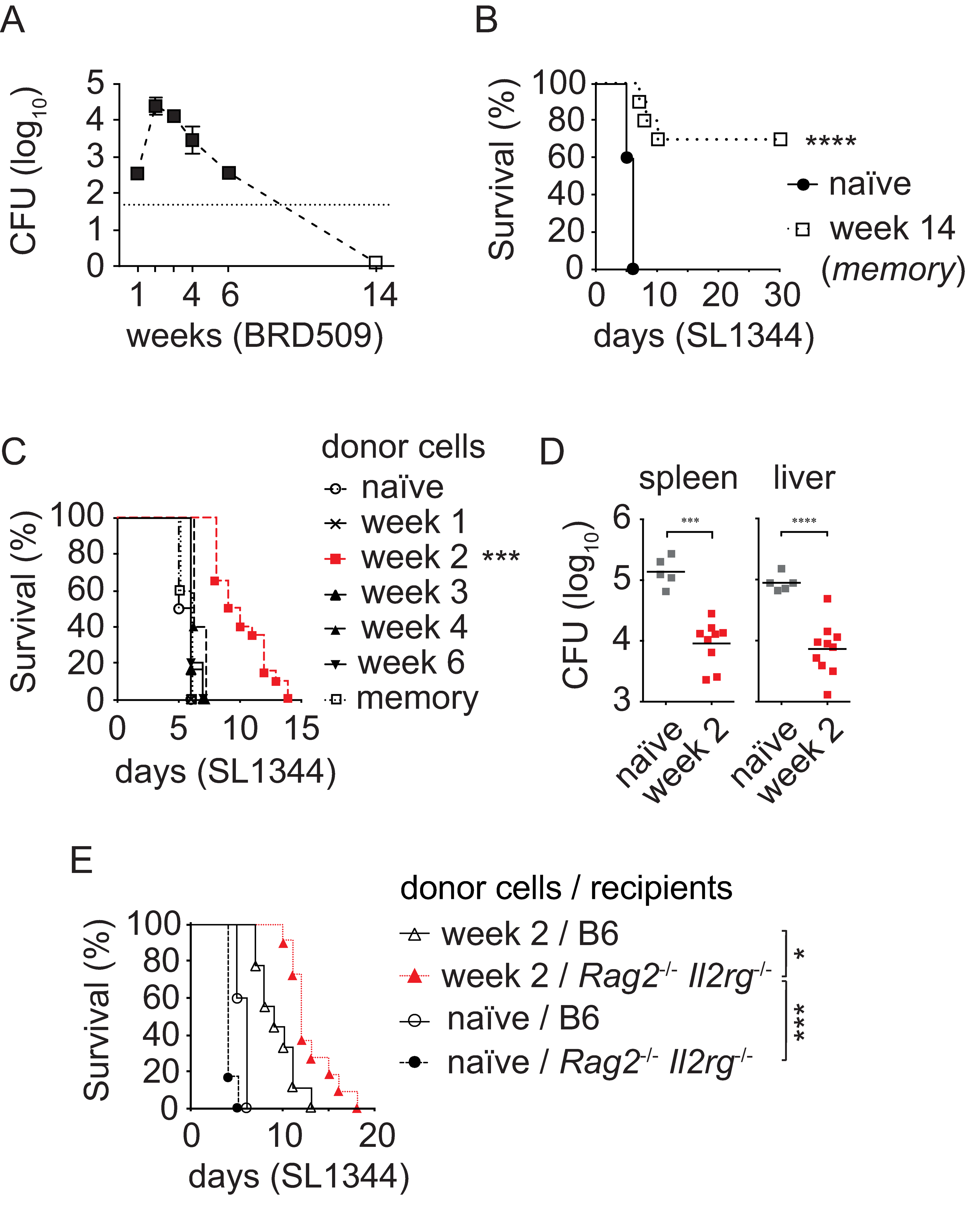

Supplement: S1 Fig — B6 mice (6–8 weeks old) were infected i.v. with 200CFU BRD509 strain. (A) Bacteria counts on solid LB media from homogenate spleen of infected mice over the course of the primary infection. (B) Survival of mice immunised with BRD509 i.v., or uninfected, and 14 weeks later challenged with 200CFU SL1344 i.v.. (C) Survival of groups of mice that adoptively received 5×10^7^ total donor splenocytes harvested at weeks 1, 2, 3, 4, 6, 14 post-single BRD509 infection and 24h post-transfer were challenged with 200CFU SL1344 iv. (D) SL1344 counts on differential, solid XLD media from spleen and liver of B6 mice recipients of week 2 or naïve donor splenocytes on day 4 post-challenge. (E) Survival of either complete (B6) of lymphocyte-deficient (Rag2-/- Il2rg-/-) mice that adoptively received 5×10^7^ total naïve or week 2 BRD509 donor splenocytes and 24h post-transfer were challenged with 200CFU SL1344 i.v.. Data is representative of (A,B,E) 2–4 and (C,D) 2 pooled independent experiments with (A) 4–8, (B) 10, (C) 5–7, (D) 5–10 and (E) 6–11 samples per group. (A) Symbols and bars represent mean and SEM, (B,C,E) symbols represent percentage of survivors, and (D) lines and symbols represent mean and individual measurements. respectively. Statistical analysis, log-rank (Mantel-Cox), multiple t-student test. ***p<0.005, ****p<0.001. (TIF) [file ppat.1010004.s001.tif]

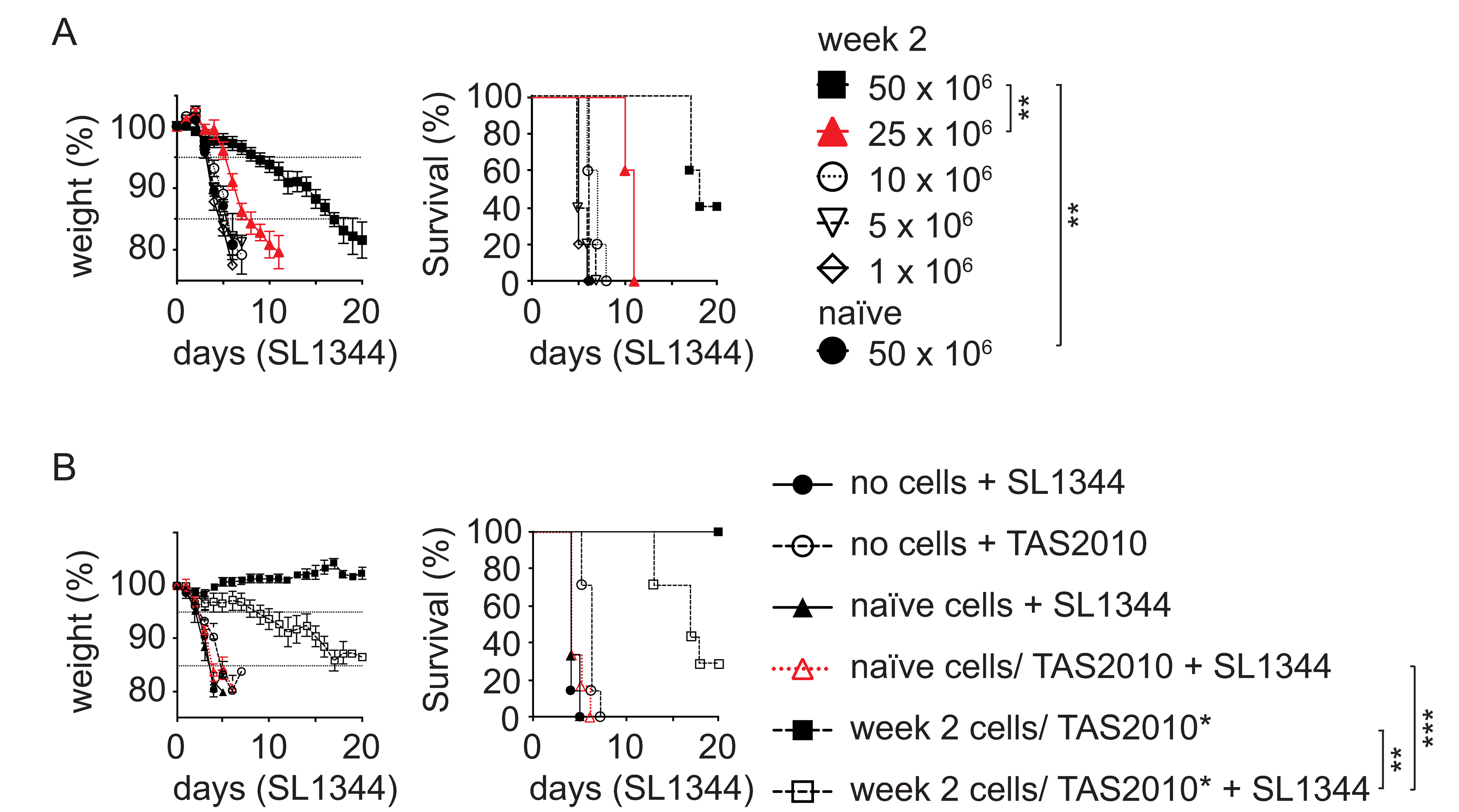

Supplement: S2 Fig — (A) Weight loss (left) and survival (right) of Rag2-/- Il2rg-/- mice that received different numbers of splenocytes from week 2-TAS2010 infected mice, and the recipients were challenged with 200CFU SL1344 24h after adoptive transfer. (B) Weight loss (left) or survival (right) of Rag2-/- Il2rg-/- mice that received i.v. 5×10^7^ splenocytes from either week 2-TAS2010 infected B6, or uninfected B6, or did not receive cells; control that were adoptively transferred ~200 CFU TAS2010 along with splenocytes (*) or injected with 200CFU TAS2010 i.v. at the time of transfer. 24h post-transfer recipients were challenged with SL1344 or left unchallenged. Data is representative of (A,B) 2 pooled independent experiments, with (A) 10 (B) 7–10 animals per group. Statistical analysis, log-rank (Mantel-Cox). **p<0.01, ***p<0.005. (TIF) [file ppat.1010004.s002.tif]

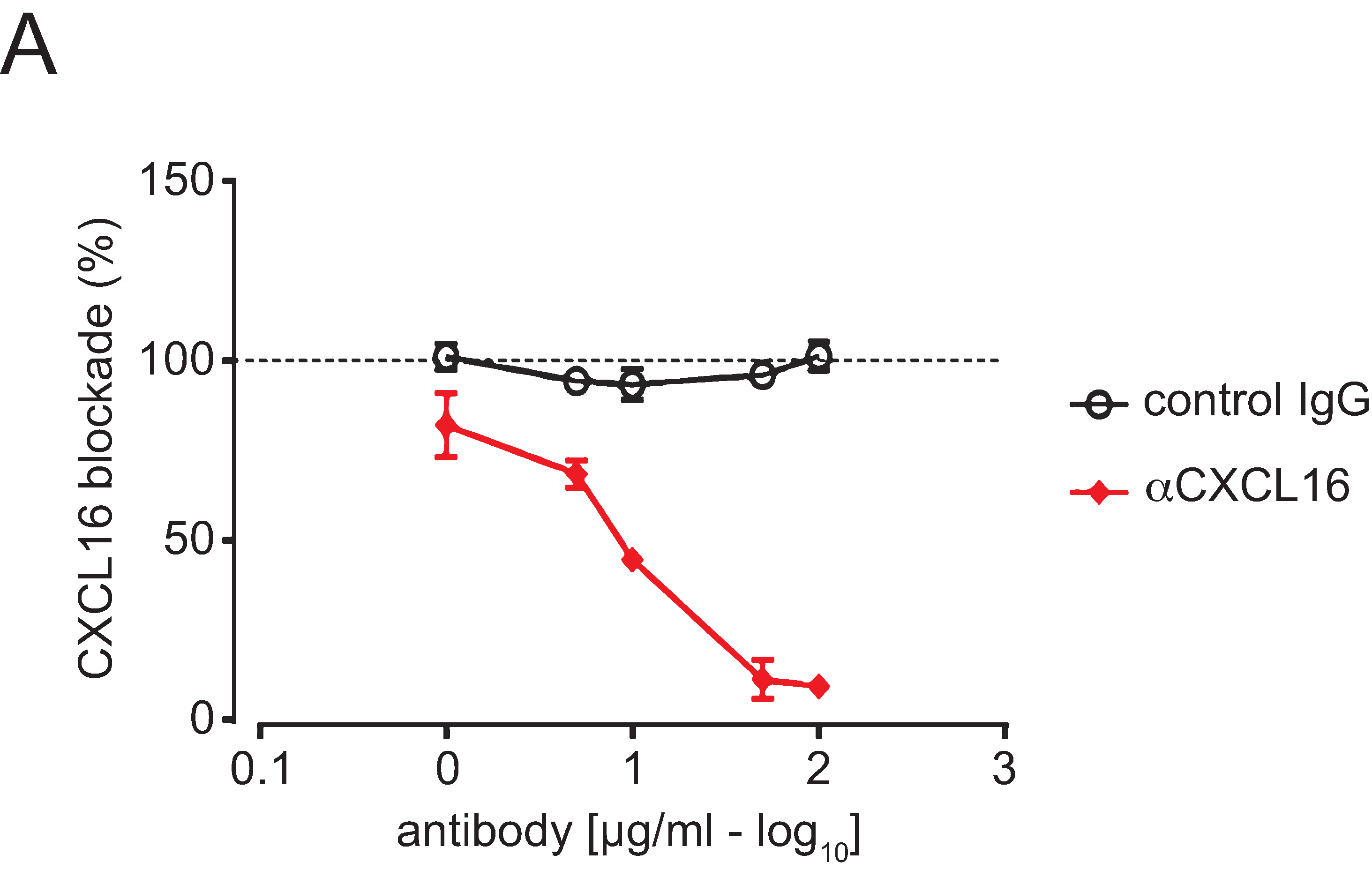

Supplement: S3 Fig — (A) Neutralization of CXCL16 was measured by the inhibition of migration of murine CXCR6-expressing B300.19 cells towards soluble CXCL16 in vitro [26]. Symbols represent mean and bars represent SEM. Data is representative of pooled independent experiments, with 3–4 samples per group. (TIF) [file ppat.1010004.s003.tif]

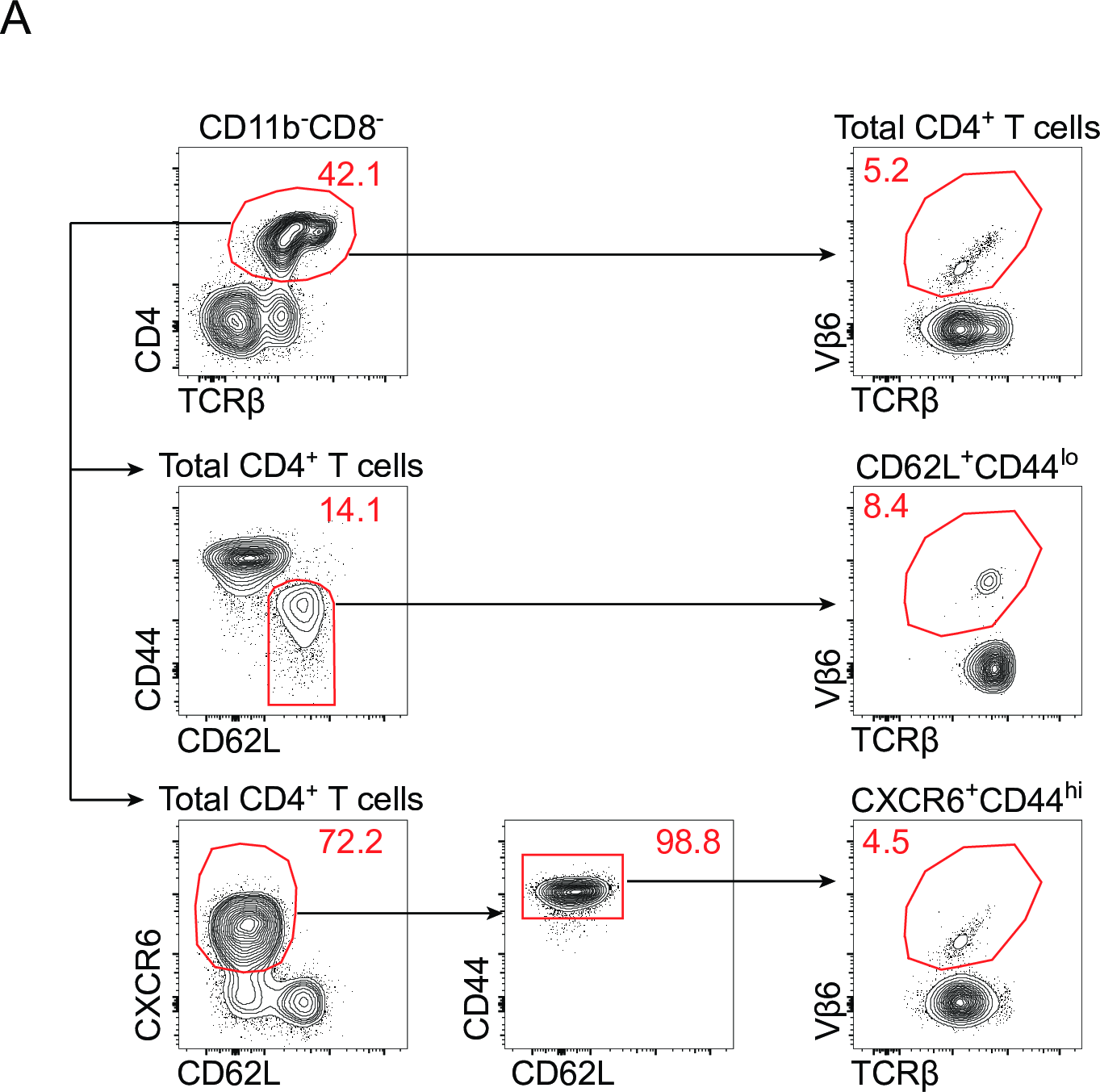

Supplement: S4 Fig — (A) B6 mice were immunised with 200CFU TAS2010 i.v. 12 weeks later, the mice were killed, the livers perfused with PBS and the cells collected. Representative FACS plots are also provided for gating on CXCR6+CD44hi and CD62L+CD44lo subsets for liver CD4+ T cells. (TIF) [file ppat.1010004.s004.tif]

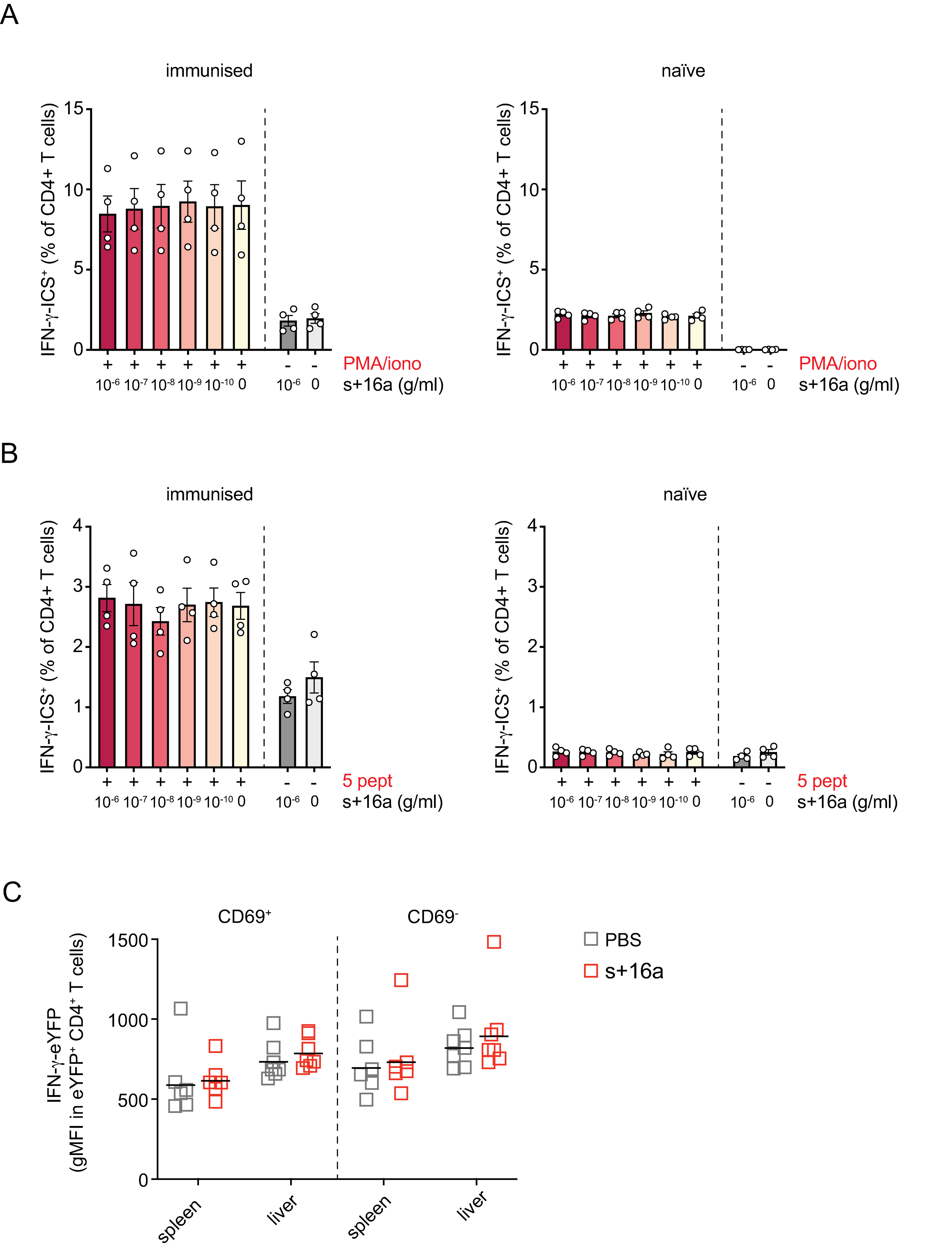

Supplement: S5 Fig — Wild type B6 mice were either naïve or infected with 200CFU TAS2010 i.v. At week 5 p.i., splenocytes were harvested and re-stimulated ex vivo in the presence of indicated concentration of s+16a nanobody at 2×10^6^ cells per well in 200μl (n = 4). The nanobody was present during the entirety of the re-stimulation protocol, either with (A) PMA and ionomycin for 4h with brefeldin A, or (B) a pool of Salmonella peptides (5pept, as per Fig 5) for 18 h with brefeldin A added in the final 4 h. At the end of the incubation period, re-stimulated cells were intracellularly stained for IFN-γ. No statistically significant difference (one-way ANOVA with Bonferroni post-tests) was observed between re-stimulated cells treated with different concentration of s+16a nanobody. (C) IFN-γ-eYFPin/in mice were infected with 200CFU TAS2010 i.v. for 12 weeks. Shown is the geometric mean fluorescence intensity (gMFI) of IFN-γ-eYFP in both CD69+ and CD69- CD4+ T cells in mice that were pre-injected with either 50μg of s+16a nanobody or equal volume of PBS 15-20min immediately prior to euthanasia and tissue collection (n = 6–8). The liver was then perfused with PBS to remove circulating cells. No statistically significant difference (Student t-test) was observed between s+16a or PBS pre-injected mice. Symbols represent data from individual mice, mean±SEM shown. (TIF) [file ppat.1010004.s005.tif]

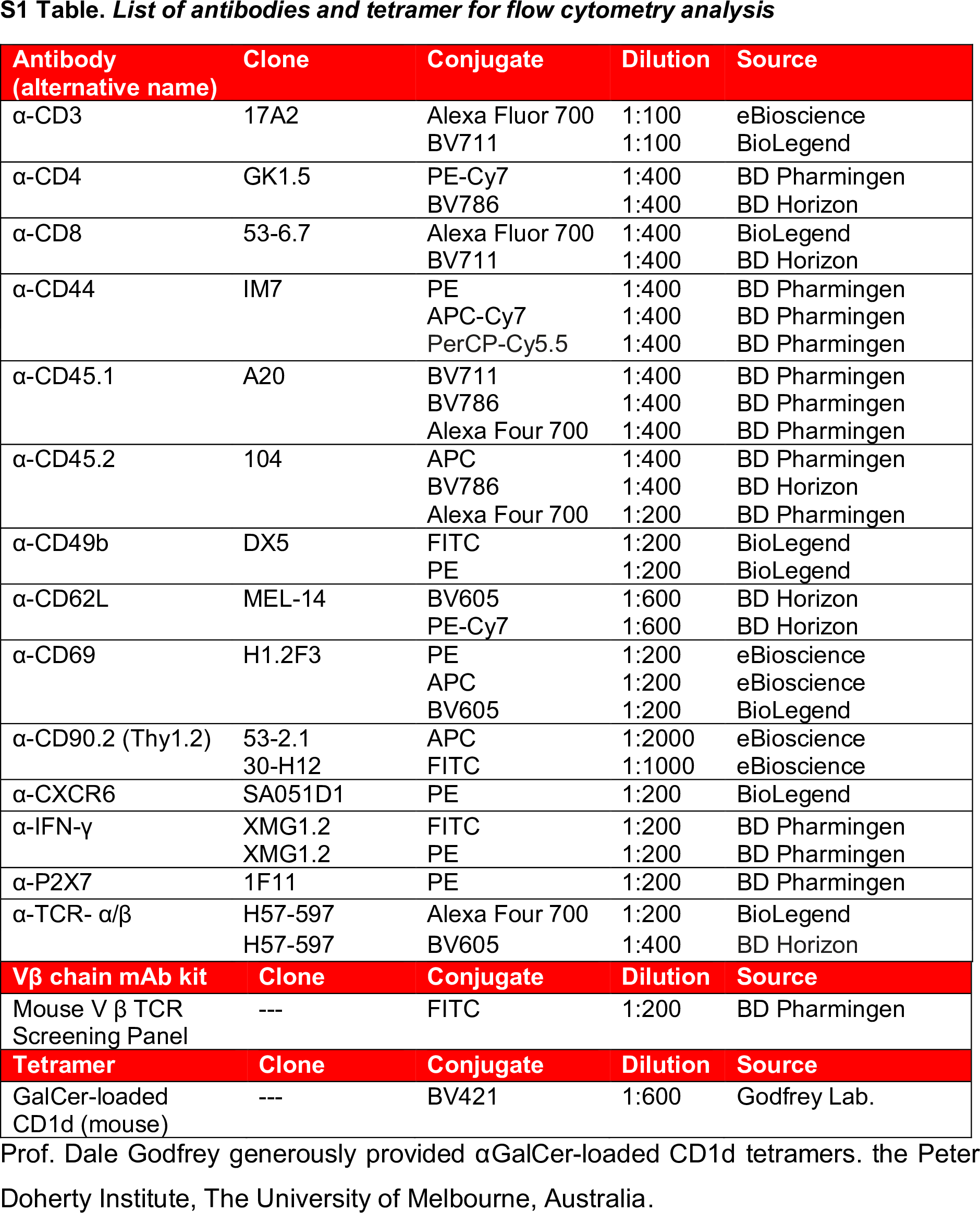

Supplement: S1 Table — (TIF) [file ppat.1010004.s006.tif]

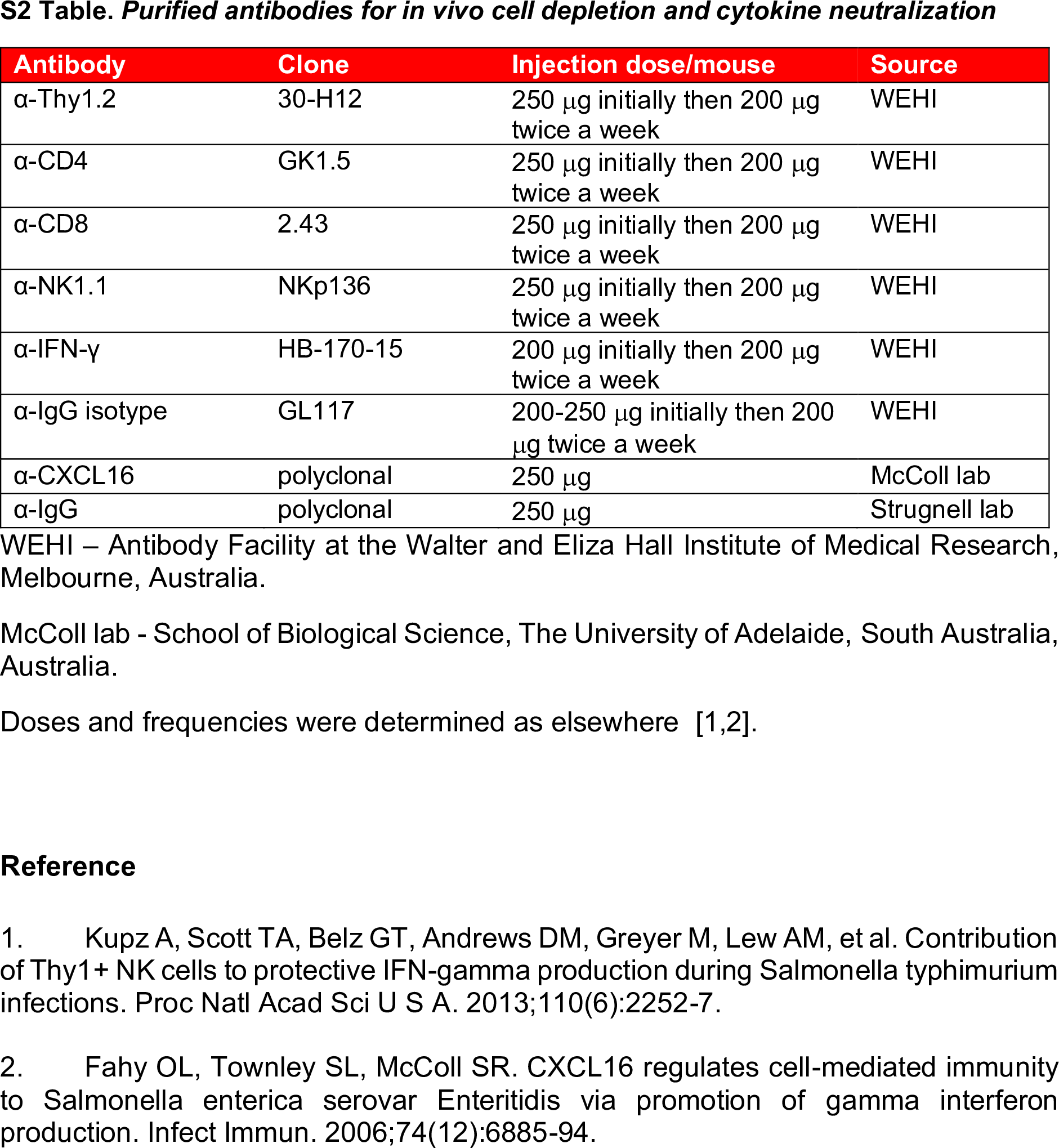

Supplement: S2 Table — (TIF) [file ppat.1010004.s007.tif]
